# Analytical performance assessment and suitability of the plasma β‐Amyloid 1‐40 and 1‐42 assays on the Lumipulse G instrument from Fujirebio to support Alzheimer's Disease clinical trials

**DOI:** 10.1002/alz.085537

**Published:** 2025-01-09

**Authors:** Magali Dupont, Philippe Frantz, Séverine Thabuis, Divya Nalayanda, Erica Troksa

**Affiliations:** ^1^ Labcorp Central Laboratories, Geneva, Geneva Switzerland; ^2^ Labcorp Central Laboratories, Indianapolis, IN USA

## Abstract

**Background:**

Alzheimer’s Disease (AD) is a major neurodegenerative disorder characterized by amyloid deposits in brain tissues and representing a continuously increasing global burden in need of disease‐modifying therapeutic options. Amyloid beta 1‐42 and 1‐40 peptides and the amyloid beta 1‐42/1‐40 ratio are hallmarks of AD and are commonly monitored in Cerebro‐Spinal Fluid (CSF) along other AD biomarkers, to support diagnosis and management of AD patients. Over the past few years, blood‐based AD biomarkers have emerged as highly relevant and more practical alternatives to CSF biomarkers, and further technical performance characterization of the associated assays would be beneficial to the AD research and medical community.

**Method:**

The Lumipulse G β‐Amyloid 1‐42 and 1‐40 Plasma assays are two‐step chemiluminescent enzyme immunoassays (CLEIA) performed on the Lumipulse G system and designed for the quantitative measurement of amyloid beta 1‐42 and 1‐40 in plasma specimens. We evaluated these two assays in a CAP/CLIA environment, utilizing CLSI guidance to set validation parameters and acceptance criteria. Linearity of results, assay precision within and between analytical runs, functional sensitivity as well as other performance parameters were evaluated in endogenous samples, neat or spiked to obtain appropriate concentrations.

**Result:**

Utilizing a set of samples from apparently healthy donors above 75 years old, we were able to show an expected reference range of 154.05 – 455.50 pg/mL and 12.57 – 42.43 pg/mL for amyloid beta 1‐40 and 1‐42, respectively. Our results support a linear measurement range of 0.57 – 3,404.60 pg/mL and 0.83 – 682.00 pg/mL for amyloid beta 1‐40 and 1‐42, respectively, hence covering appropriately the expected reference range of the healthy population and allowing to capture abnormally elevated or depressed concentrations. For amyloid beta 1‐40, experiments revealed CV% ranging from 3.3 to 3.7% for intra‐ and 5.5 to 7.0% for inter‐assay precision. For amyloid beta 1‐42, CV% ranged from 3.4 to 5.4% for intra‐ and 5.8 to 8.8% for inter‐assay precision.

**Conclusion:**

Overall, these assays demonstrate technical robustness and reliability to support clinical trials for AD in the plasma matrix, which can offer a less invasive, yet valuable test to enrich for amyloid positivity in neurodegenerative clinical trials.

